# Book Reviews

**Published:** 1822-01

**Authors:** 


### Monthly Medical and Scientific Bibliography.

Vexat censura . . . . . . Corvos.

### I. GREAT BRITAIN.

**Art. I.**—*Enchiridion; or a Hand for the One-handed.* By George Webb De Renzy, Captain, H. P. 82d Regiment. pp. 57. Sams, London. 1821.*

THE author, having lost his right arm at the battle of Vittoria, and having, consequently, experienced serious inconveniences, turned his attention to the means of making the best of his misfortune; and succeeded in inventing, or improving, a set of instruments, which he

* *Our Subscribers, and the Profession in general, may be supplied with any of the Foreign or English Publications noticed or reviewed in this Journal, on the shortest notice, by addressing a line to J. Souter, 73, St. Paul's Churchyard; by whom this Journal is regularly delivered to the Subscribers on the first of every month.*

has offered to the notice of his Royal Highness the Commander-in-Chief, in hopes of enabling some of the numerous individuals who may be in similar circumstances with himself, to derive that benefit from the use of them which he has done. The articles described as belonging to the one-handed apparatus are the following:—" 1. Wash-hand tray, complete. 2. Ivory vice, with ball and sockets. 3. Shaving box. 4. Lead cushion. 5. Syringe. 6. Nail file. 7. File-holder. 8. Boot-hooks. 9. Silver egg-cup. 10. Steel egg-holder. 11. Pen-knife. 12. Quill-holder. 13. Pen-nibber. 14. Ruler. 15. Steel vice. 16. Hat-stick. 17. Knife and fork. 18. Nut-crackers. 19. Card-holder. 20. Case for the instruments."

---

Art. II.—*Dissertatio Medica Inauguralis de Cordis Palpitatione; quam, pro Gradu Doctoris, summisque in Medicina Honoribus ac Privilegiis rite et legitime Consequendis, eruditorum examini subjicit* Carolus Locock, Anglus, Reg. Med. Soc. Edin. Socius.

Dr. Charles Locock very properly observes, in the introduction to his essay, that, at his age, and in default of much personal experience, " aptius est ex aliorum observatione, potius quam ex sua, quæ sit dicturus, deducere." At the same time, we cannot admit that he is altogether incapable of supplying us with original observations, either on the subject of his present dissertation, or upon any other, connected with medical science, of which he may choose to treat. A person of mature reflection, however tender his years may be,—the son of a much-esteemed and excellent practitioner,—the pupil of Brodie,—and a constant attendant on the lectures of the first professional characters in this metropolis,—one who, after having frequented the London hospitals, starts in search of ulterior knowledge, through France, Switzerland, and Italy, where, for upwards of twelve months, he enjoys the advantage of friendly, and almost daily, lessons from Vaccà, Morelli, and Catellacci;—and who, after all this, sets himself down for another long period of academical instruction in the bosom of the northern Alma Mater,—must certainly have acquired the right of imparting to the public something beyond a mere compilation. We shall, therefore, hail with pleasure the accomplishment of Dr. Charles Locock's prospective promises:—" Quod si eadem postea provectiore ætate prosequar, de iis rebus melius judicare ex experientia licebit, quæ in juventute rationi solum consentaneæ esse videntur."

The dissertation before us contains a pretty extensive medical history of the disease mentioned in the title-page, and embraces several apt considerations on its causes, diagnosis, prognosis, and treatment. Throughout, Dr. Charles Locock has made use of the information he has derived from the best authors, who have written on the heart and its morbid affections; nor can he be accused, like a recent English author on diseases of that organ, of having neglected to refer to by far the best work on the subject. Dr. C. Locock mentions both Laennec's (not Læннac) treatise and his *stethoscope*.

We were happy to find Dr. C. Locock on our own grounds, when, in speaking of palpitations of the heart produced by suppressed discharges, he, the worthy child of a good practitioner, and no visionary theorist, recommends a perpetual issue as part of their treatment. Modern physicians have unjustly neglected this remedial agent in chronic diseases.

---

Art. III.—*The Physician's Guide; being a popular Dissertation on Fevers, Inflammations, and all Diseases connected with them; comprising Observations on the Use and Abuse of Blood-letting, Mercury, Cathartics, Stimulants, Diet, &c. &c. &c.* By Adam Dods, m.d. pp. 320. Worcester, 1821.

This is a large book, which we intend to dispatch in a very few words. The fact is, that we are really puzzled what to do with it. We know nothing of the author; and we must conclude that he is a respectable man of experience, writing for the improvement of his brethren,—and particularly of those who do not happen to be equally as clever as himself. If so, we think he has adopted (to say the least) an unwise method;—namely, that of throwing out general anathemas against the majority of the profession, and of holding them up as HALF PRACTITIONERS, "*unacquainted with the various stages of diseases, because unacquainted with the different branches of the profession;*"* as if the knowledge of chemistry, botany, and veterinary surgery, for instance, were necessary to understand the cold, the hot, and the sweating stage of fever! Again, Dr. Dods professes himself an admirer of Dr. Jackson and Dr. Armstrong; for neither of whom, we confess, we ever entertained one-third of that ill-judged reverence which some of our contemporaries, too hasty in their admiration, professed to entertain for them some years ago; but which has, since, sunk into a prudent silence. The great secret of Dr. Armstrong's ephemeral reputation founded on his work on Typhus; and the subsequent want of consideration paid to the principles it contains, lies in a nut-shell:—when that gentleman wrote, we were all *higgledy-piggledy*† on the subject of fever. The remains of Brunonism had been nigh making us forget that there was such a thing as the lancet. Dr. Armstrong saw this, and urged blood-letting (a lucky hit,) in a species of fever, which, though bearing a new name, had long been known, and had, long before, been treated by venesection. The result of this change of practice in this country was, of course, beneficial to humanity. Unfortunately, however, the remedy was somewhat indiscriminately applied to a multitude of other cases, and the assistance of strong cathartics was called in, to spoil the whole; so that the mortality, instead of diminishing, once more appeared to be on the increase. Now, Dr. Dods has undertaken to prove that this system is correct; and, in his "cure of fever,"‡ he tells us, "that we should lose no time to lessen the cold in the cold stage, the heat in the hot stage, and not await the sanative process of nature." These things may all be very correct; but surely the means by which Dr. Dods proposes to

---

* Page 87.

† We trust the *purists*, or, as a contemporary of ours calls them, the "Athenians," will allow us the use of this cant word for once. It serves our purpose, admirably, on this occasion.

‡ Page 86.

carry them into effect, are not the best calculated to insure success. On the whole, we think Dr. Dods writes under the influence of some hidden motive, the nature of which we pretend not to divine: but, when our readers shall have perused the following passage, which we select, at random, from that gentleman's work, they will agree with us, that sentiments like those it contains must be dictated by other feelings than those for the mere advancement of science.

"Inflammation of the peritoneum often occurs in women after delivery, and is occasioned by the same causes which occasion uterine inflammation. In January last, another surgeon-apothecary of this city, who has been in practice more than thirty years, had two of his patients, in one week, die of diseases of the puerperal state. The one, being a poor woman, had no consultation. The other was rich, and had a consultation. Now, which of these two unfortunate females had the *best chance* of recovery? A superficial and unreflecting observer will immediately assert, that it was the one who had the consultation. Let us then examine the merits of this; for it is of vital importance to the public that it should be explained. It appears, this surgeon-apothecary and accoucheur, together with six or seven others of the same class, pronounced, in the Worcester newspapers, in the year 1805, themselves incapable to conduct the treatment of diseases in their complex state, but that they were to have the management of them only in the first instance, when they were supposed to be simple; and, on their becoming dangerous, or were made so, a physician or a surgeon (as the case may be medical or surgical, was to be consulted; and that they were to decide when this was necessary, although, from their own declaration, they could not recognize the nature of the disease and its various stages. But, in this case above alluded to, when surgical assistance and decisive measures were really necessary, this surgeon-apothecary neglected to consult any one; and, after delivery, no consultation was held until a few hours before the patient expired. The physician (who was the same learned Doctor as alluded to in the foregoing case of inflammation of the bowels,) declared that he was called in too late. Of what use, then, was this consultation? And, supposing this physician had been called in sooner, what service could he have rendered, when it is well known that he has no practical knowledge of diseases of the puerperal state, his information being merely theoretical? He may probably have read of them, and he may talk of them with much volubility, (for with copiousness of speech the learned Doctor is said to be at least profusely, if not profoundly, gifted;) but at the bed-side of the patient he could not, for want of practical knowledge, distinguish one disease from another, or trace their rise and progress: he, therefore, was a very unfit person to be consulted on such a serious occasion."

This is but a poor specimen of what a "Physician's Guide" should be.

---

Art. IV.—*Twelve Essays on the Proximate Causes of the Material Phenomena of the Universe: with illustrative Notes.* By Sir Richard Phillips. 12mo. pp. 458. London, 1821.

As the expectations of Sir Richard, in presenting us with a new system of physics, are very moderate, with regard to the sort of reception which it is likely to meet with in the world,* we shall not quarrel with him for entertaining, as he does, so mean an opinion of scientific men in general; since it would be rather more than feeble humanity can boast

* "The author entertains little expectation that his doctrine will be received in the spirit of liberality."

of, to expect that a class of people, roughly handled for their supposed errors, should applaud the man from whom they receive their castigation. Sir Richard is, beyond doubt, a most extraordinary man; and, although it has, once, been the fashion (we trust it is not so at present,) to smile at every thing he either said or did; it is nevertheless true that many hundred individuals, of far inferior talents to his, have had their encomiasts for a few good trifles, some scores of which are to be met with in all the writings of Sir Richard. Nor is the present work destitute of pearls. Extravagant as some of the author's ideas on the economy of nature may appear to the world, and unsupported by mathematical demonstration, which in our days is justly considered the only road to the attainment of truth, there are facts, observations, and reflections, in the present volume, which it will be well to consider and to profit by. With the subject of the first nine essays we have nothing to do.

"Essay 1. Strictures on the System of Physics taught by Kepler, Newton, and their followers.
" 2. Of the true Universal Cause, and of its Properties, Accidents, and Species.
" 3. On the Cause of the Fall of Bodies to the Earth.
" 4. On Universal Space, its Contents, and Mechanical Connexions.
" 5. On the Solar System, and the Planetary and Cometary Phenomena.
" 6. The Theory of Tides.
" 7. On the Changes which take place on the Earth's Surface, from its Motions as a Planet.
" 8. On Atomic Motion, and its Phenomena.
" 9. On Combustion, Light, Colours, and Sound."

The Essay on Electricity, Galvanism, and Magnetism, which follows, is somewhat more connected with our business. Sir Richard thinks that superstition, and the love of the marvellous, have revelled in great luxury of variety and absurdity, in the existing theories of the various phenomena called electrical. "There is, in truth, *no such thing as an electrical fluid per se;* and all electrical appearances are mere mechanical local accidents of passive matter, temporarily disturbed by the causes which generate electrical phenomena." Sir Richard explains all electrical phenomena on chemical facts, and asserts that the supposed attractions and repulsions observed in electrical experiments are simple mechanical effects. Thus far he is not singular in endeavouring to identify the electric and chemical forces; and it will not be long ere we shall see a successful attempt made to assimilate these forces to those attributed to magnetism.

In the 11th Essay, Sir Richard, we fear, has gone rather beyond his depth. His "general views of animated and vegetative nature" are singular, and that is all. They are certainly not fair inductions from facts; not even from those that he has himself brought forward. The truth of the matter is, that Sir Richard, like a few other writers of the present day, pretends that animal organization, and the examination into its powers, combinations, and effects, as well as its phenomena and uses, form the proper business of metaphysical research; in which, they are mistaken. There is a curious calculation in this essay, (purely inventive, of course,) which is intended to show that, supposing the powers of reason and retention to commence at five years of age, the year that passes from seven to eight will be one-half of all past existence, and will, consequently, be of great apparent duration; but the year that passes from twenty to twenty-one will be but a fifteenth part of all past existence, and will, therefore, in its impression on the mind, be greatly less than the former year! Upon this assumption, Sir Richard has framed the following table:

"1 to 5 ——— childhood,
5 to 10 equal to *sixty* months,
10 to 15 equal only to forty-five months,
15 to 20 equal to thirty-five,
20 to 25 equal to thirty,
25 to 30 equal to twenty-seven,
30 to 35 equal to twenty-five,
35 to 40 equal to twenty-three,
40 to 45 equal to twenty-one, nearly,
45 to 50 equal to twenty,
50 to 55 equal to nineteen,
55 to 60 equal to eighteen months."

---

Art. V.—*A Series of Questions and Answers, for the Use of Gentlemen preparing for their Examination at Apothecaries' Hall; with copious and useful Tables annexed.* By Charles Mingay Syder, Licentiate of the Honourable Society of Apothecaries, &c. &c. &c. 18mo. pp. 128. Highley, London. 1821.

It is a common complaint, now-a-days, that there are more meddlers in physic than real physicians. An explanation of this fact may be readily found in the endless number of Manuals, Vade-mecums, Guides, Series of Questions and Answers, Gazettes of Health, and Journals of Popular Medicine, that are daily published for the edification of the ignorant. Do what we may, there can be no *royal road* to the knowledge of physic. Your learning-made-easy books will teach people to prate about what they know nothing of, or enable them to get through a little collegiate sifting,—but that's all. Physic, the real genuine art, they can never teach. We dare say, that Mr. Charles Mingay Syder is a well-disposed person, who conscientiously sat himself down with the intention of rendering what he may consider a real service to the long train of pupils destined to enter the Hall, and of smoothing their road to Bridge-street; but, in reality, we are sure that his book is calculated to do no good. At the same time, we are free to confess that, as questions and answers, the various points of medical science treated of in this little volume are generally accurate, clear, and precise. As *memoranda*, therefore, to a person already well versed in medicine and pharmacy, we doubt not but they may prove of service.—The *first* series of questions turns on chemistry, medeography, and pharmacy.—The *second* on the practice of physic, the whole of which is skilfully condensed into twenty duodecimo pages; and yet the old whigs are for ever exclaiming, "Ars longa, vita brevis!" We should now be tempted to retort, "Vita brevis, sed ars brevissima."—The *third* series is upon the anatomy of the viscera, physiology, &c. Why these latter subjects are to be inquired into after the practice of physic has been gone through, we know not: unless, indeed, Mr. Syder means to insinuate that gentlemen examined at the Apothecaries' Hall may practise physic before they know any thing of anatomy and physiology: a fair induction, verily. If this be the order in which examinations are carried on at the Hall, we can only say that the examiners begin where they should end, and act precisely the reverse of what obtains at the Royal College of Physicians, where the examinations stand thus:—First day, latinity and the knowledge of Greek, anatomy and physiology; second day, latinity, the knowledge of Greek, and practice of physic; third day, medeography and chemistry.—There are several useful tables attached to this small volume.

---

### II. FRANCE.

ART. VI.—*Rapports et Consultations de Medicine legale, recueillies et publiées par* I. RISTELHUEBER, D.M. Médecin en chef à la Hospital civil de Strasbourg, &c. &c. 8vo. pp. 172. Paris, 1821.

THIS volume contains much valuable information on various subjects of juridical medicine. The most important question discussed in it, however, is one arising out of a particular article in the Civil Code of France, which enacts "that a contract for an annuity on the life of a person who died the same day on which the contract was signed, or within twenty days from the passing of the contract, becomes null and void." This question, it is evident, can only be decided on the report of a *medecin legiste*, particularly in such a country as France, where all these matters are the never-failing subject of much formality, *buro-cratic* influence, and the display of unnecessary erudition. The case in point, and debated by Mr. Ristelhueber, is one which attracted great notice at Strasbourg some years ago. A Mr. Fried sold, on the 11th of March, 1809, a large sum in the funds for an annuity on his own life. He was, and had been, at the time of the bargain, afflicted by hemiplegia for ten years, in consequence of an apoplectic stroke; and died on the second day from the signing of the documents. The question, therefore, is, whether Mr. Fried, on the day on which he signed the papers, was, or was not, already, under the influence of the disease to which he fell a victim thirty hours afterwards. The question was debated with much talent on both sides; and the volume before us contains the various reports, opinions, and arguments, written on the occasion.

In the second section of his volume, Mr. Ristelheuber has presented us with several cases of infanticide, in which there are fresh proofs in support of the validity of the evidence furnished by pulmonic docimasy in trials for that heinous crime.

A case of poisoning by arsenic is contained in the third section, from which it appears that the deleterious qualities of that oxide were considerably weakened by its association with some articles of food, in which it had been administered. In one of the chemical experiments made to ascertain the presence of the arsenic, this substance was obtained in a metallic state.

Art. VII.—*Defence des Médecins Français contre le Docteur Broussais, Auteur de la Nouvelle Doctrine Medicale; ou, Lettres Medicales à M. Broussais suivies d'un Traité complet de Médecine pratique d'après la Doctrine la plus généralement reçue en France.* 1re. livraison. Par S. P. Authenac, Médecin en chef de plusieurs Hôpitaux. 8vo. pp. 176 and 163.

Art. VIII.—*Histoire de quelques Doctrines Medicales, comparées a celle du Docteur Broussais.* Par Mr. Fodera, d.m. pp. 233. Paris, 1821.

Here is Monsieur *Tonson* again!—Really this is too much; and we dare say Dr. Broussais himself finds it so by this time. It was to be expected that the French physicians would not tamely submit to the accusations brought against them by that reformer; and that his pretensions to novelty and invention would be freely canvassed and investigated. Both these events have taken place. Dr. Authenac, in a series of letters, nine in number, addressed to the head of the Physio-Pathological School, defends the generality of his fellow-practitioners in France from the attacks of that chief; while Signor Foderà, anxious to assert the claims of several Italian physicians to the originality of all the good things that Broussais has proclaimed, exposes, in another volume, the numerous plagiarisms of which he has been guilty.

Dr. Authenac treats of modern classifications, of the definition and description of diseases, and of those facts on which both are founded. His fourth letter is on medical constitutions; the fifth, on inflammatory fevers; the sixth, on bilious fevers; the seventh, on adynamic and ataxic fevers; the eighth, on intermittents; and the ninth is dedicated to the recapitulation of all the preceding observations. These letters are followed by the first part of a Medical Nosography, proposed by Dr. Authenac.

Signor Foderà is much more gentle, though by no means less convincing, in his attack on Broussais. He has contrasted the writings of Baglivi and Rega with those of the Parisian professor, and proved that the latter has made the most of his erudition, in appropriating to himself the neglected discoveries of others.

---

Art. IX.—*Recueil de Memoirs de Chirurgie.* Par le Baron Larrey. 8vo. pp. xiv. 320; four plates. Paris, 1821.

The principal memoir in this volume is on Moxa, containing the history and use of that remedy; the mode of applying it; and the diseases in which it has been employed with success. The complaints in which the Baron himself, or his friends, have had recourse to this surgical application with good effects, are the following:—1. Morbid affections of the faculties of sight, smell, taste, hearing, and voice. 2. Paralytic affections of the muscular system. 3. Different species of palsy. 4. Organic diseases of the viscera in general. 5. Diseases of the chest. 6. Old catarrhal affections and chronic phlegmasiæ of the pleura. 7. Pulmonic phthisis. 8. Chronic and organic diseases of the abdominal viscera. 9. Rachitis. 10. Sacro-coxalgia. 11. Femoro-coxalgia.—There are nine cases in illustration of the benefit derived from the application of moxa in diseases of the spine; and seven in favour of the same remedy for the cure of femoro-coxalgia.

The other papers contained in the volume are two memoirs on the Seat and Effects of Nostalgia; one on Fracture of the Neck of the Femur; and two essays, on the Properties of the Iris, and on Wounds of the Intestines.

---

Art. X.—*Additions à l'Anatomie Generale de* Xavier Bichat, *pour servir de Complement aux Editions en quatre volumes.* Par P. A. Beclard, Professeur d'Anatomie et de Physiologie à la Faculté de Médecine de Paris. 8vo. pp. xvi. 392; with a Portrait of Bichat. Paris, 1821.

In our Essay on the Progress of Medical Science, inserted in the present Number, we have alluded to this important volume. A book of notes and additions is not susceptible of being analyzed. One may easily judge of the necessity, as well as the importance of some such undertaking as that to which Mr. Beclard has consecrated a portion of his time, from the immense progress which every branch of anatomy has made since Bichat published his first edition in 1801. Prof. Pinel and Mr. Laennec had engaged to co-operate, since the year 1818, with Mr. Beclard, in the compilation of the present volume; but, the state of ill health of both those eminent physicians, at that time, having precluded them from fulfilling their engagements, Mr. Beclard has alone carried the plan into effect. To render the volume complete, Mr. Beclard has spared no pains. Independently of all the new facts for which we stand indebted to the French anatomists and physiologists, and, among them, to Mr. Beclard himself, he has laid contributions on the principal works published in this country and in Germany; having consulted the *Treatise on General Anatomy,* by Meckel and Gordon; the *Prodromus of General Anatomy*, by Mascagni; the *Histology* of Meyer; besides those of Bock, Baillie, Otto, Cruveilher, and Laennec.

---

### III. AUSTRIA.

Art. XI.—*Practische Abhandlungen über die vorzuglichern Krankheiten des Kindlichen Alters; or, a Practical Dissertation on the principal Diseases of Children.* By L. A. Goelis, m.d. first Physician to the Institution for Sick Children at Vienna. 8vo. pp. xii. 312.

The first edition of this work was printed in 1815; and, from the favourable reception it met with, the author has found it necessary, again, to offer it to the public, in an enlarged and a more perfect form. The author, who must be well known to our English readers, from the translation of his work on Hydrocephalus, lately published by Dr. Gooch, informs us, in the present edition, that the observations and opinions which he offers to the public are the results of his practice during the period of twenty-three years at the Institution for Sick Children in Vienna, where, in the course of that time, 130,594 patients were admitted. This appears manifest from an accompanying table, in which the ages, as well as the diseases, of the children are marked. Our limits do not allow us to dilate on the merits of this publication; but, from the known experience and talents of its author, as well as from the distinguishing marks of favour with which this volume has been received on the continent, we hesitate not in recommending it to the attention of our readers.

---

**Art. XII.**—*Armamentarium Chirurgicum Selectum.* By F. X. Rudtorffer, Professor of Surgery at Vienna. 4to. pp. 582; 30 plates.

This is by far the best collection of engravings of surgical instruments. The author divides them into two great classes: 1, general, and 2, specifical; each of which he has subdivided into, *a*, cutting, and *b*, blunt instruments. From a knowledge of this arrangement, it is easy to conceive how the author has treated his subject. The drawings are most accurately executed, and the description of the instruments is, thereby, rendered more easily intelligible.

---

### IV. PRUSSIA.

**Art. XIII.**—*Commentarius de Phlegmatia alba dolente.* Auctore I. L. Casper, m. & c.d. 8vo. pp. 68.

The author reviews all the writers on this subject, both ancient and modern, beginning from Mauriceau, to whom he attributes the first description of the disease in 1718. He mentions the works of Puzos, Levret, David, Callisen, Henze, and White, and many of the more recent writers. Out of 2000 lying-in women, Dr. Casper thinks one, only, is affected with swelled-leg. This disease generally makes its appearance between the eleventh and fifteenth day after delivery, attended with pain in the loins and other symptoms of fever. The author divides it into acute and chronic. In the first stage of the disease he recommends the antiphlogistic plan, and advises the use of electricity.

---

### V. BAVARIA.

**Art. XIV.**—*Handbuch zur Erkenntniss und Heilung der Kinderkrankhesten, &c.; or, a Manual towards the Knowledge and Treatment of the Diseases of Children.* By Adolphus Heuke, m.d. Professor of Therapeutics in the University of Erlangen. Third edition. 2 vols. 8vo. pp. 486, 295.

The rapidity with which this work has gone through two editions, bespeaks its value. The immediate object of the author was to supply junior practitioners, as well as those who are more experienced, with a convenient manual, to serve them as a guide in the management and treatment of infantile complaints; in which he seems to have completely succeeded. We would recommend to some medical practitioner, versed in the German language, to present the English public with a translation of this valuable work, which we feel confident, would amply repay him for the trouble.

We have selected the above work for our notice, as well as another on the same subject, mentioned under the division of *Austria*, purposely with a view of calling the attention of the British public to the necessity of placing ourselves on a level with our continental brethren in the knowledge and treatment of infantile diseases. That this branch of medical practice has been sadly neglected amongst us, is a fact admitting of no contradiction; and, while numerous and valuable works on the subject are being published both in France and Germany; and extensive institutions for the reception of sick children are to be met with in almost every capital and large city in Europe—Great Britain, till lately, could boast of no establishment for the exclusive treatment of infantile diseases. It is to be hoped that the foundation of the Universal Dispensary, by Dr. Davies, and of the still more extensive charity, called the Royal Metropolitan Infirmary for Sick Children, (where, in the course of the first year only, upwards of six thousand sick children were treated by the medical officers,) will lead to beneficial results and praiseworthy imitations.

---

### VI. SAXONY.

Art. XV.—*Pharmacopœa Saxonica.* By Leonhardi, Physician in ordinary to the King. 8vo. pp. 420. Dresden, 1820.

This work, published by royal authority, is reputed by the German critics one of the best of its class. The purity of its latinity, as well as the accuracy of its preparations, is said to be worthy of the best encomiums.

---

### VII. ITALY.

Art. XVI.—*Sull' Ernia del Perineo.* Memoria di Antonio Scarpa, Professore emerito e Direttore della Facoltà Medica dell' Università di Pavia. Five plates. 8vo. 1821.

Scarpa was the first, we apprehend, who spoke in support of the assertion of Chardenon, of his having met with an hernia in perinæo. Chopart and Dessault doubted the possibility of such a displacement of the intestines; and Sir Astley Cooper, we believe, while he admitted that a portion of the intestine may be pushed towards the very lowermost part of the pelvis, denied that it could proceed so far as to form a prominent hernial tumor in perinæo. The new work of Scarpa, which we are the first to announce to the English public, puts this question at rest. This illustrious anatomist and operative surgeon, having, occasionally, had under his treatment a patient afflicted with perinæal hernia, for which he had recommended the constant use of a particular truss, has profited of the opportunity, afforded him of examining the parts by the death of the patient, who fell a victim to pulmonary consumption, after having been for nine years afflicted with the hernia in question. The results of the dissections, carefully conducted, form the subject of the five tables which accompany the memoir, and which were wanting to complete the anatomico-chirurgical history of hernia.

VIII. SPAIN.

**Art. XVII.**—*Exposicion del Merito y Premio de la Medecina comparado con el de las demas Sciencias y otros ramos del Estado, en el año de* 1820. By Dr. Pedralbes, Physician extraordinary to the King. (From the Decadas Medico Quirurgicas, published in Madrid.)

A very accurate idea may be formed of the wretched condition of physicians in Spain, from the *contents* of this curious paper,—a sort of address, or petition, to the Cortes, by the writer whose name stands at the head of this article. It appears, from Dr. Pedralbes' statement, that practitioners in medicine have hitherto, and are at this present moment, for aught we know, considered incapable of filling any civil situation; that they enjoy no rank in society; that the lowest clerk in office takes precedence of them; that the faculty, to which they belong, is always reckoned the last in all universities; that they are poor, and receive but the paltry sum of a *pezzeta* (two reals, or one-tenth of a dollar,) for their fee; and that, when filling any public medical office, their salary is wholly disproportionate to that paid to other public officers,—the principal physician of the Board of Health having a salary only of six hundred dollars to live upon. Dr. Pedralbes, of course, urges the necessity of a reform in all this, *and the petitioner will ever pray, &c.*: and so do we pray, most heartily, that we may never witness such things.

---

